# Gene expression analysis of toll like receptor 2 and 4, Dectin-1, Osteopontin and inflammatory cytokines in human dental pulp ex-vivo

**DOI:** 10.1186/s12903-022-02621-4

**Published:** 2022-12-03

**Authors:** Arshad Hasan, Talat Roome, Mohsin Wahid, Shazia Akbar Ansari, Hira Akhtar, Syeda Neha Ahmed Jilani, Amber Kiyani

**Affiliations:** 1 Department of Operative Dentistry, Dow Dental College, Dow University of Health Sciences, Baba-E-Urdu Road, Karachi, 74200 Pakistan; 2 Department of Pathology, Section Molecular Pathology, Dow International Medical College, Ojha Campus, Gulzar-E-Hijri, Karachi, Pakistan; 3 Dow Institute for Advanced Biological and Animal Research, Dow University of Health Sciences, Ojha Campus, Gulzar-E-Hijri, Karachi, Pakistan; 4 Department of Pathology, Dow International Medical College, Dow University of Health Sciences, Ojha Campus, Gulzar-E-Hijri, Karachi, Pakistan; 5 Dow Research Institute of Biotechnology and Biomedical Sciences, Dow University of Health Sciences, Ojha Campus, Gulzar-E-Hijri, Karachi, Pakistan; 6 Department of Oral Pathology, Dow Dental College, Dow University of Health Sciences, Baba-E-Urdu Road, Karachi, 74200 Pakistan; 7 Department of Oral Medicine and Diagnosis, Islamic International Dental College, Riphah International University, 7th Avenue G-7/4, Islamabad, Pakistan

**Keywords:** Dectin 1, Irreversible pulpitis, Th1 cytokines, TLR-2, TNF-α, Osteopontin

## Abstract

**Background:**

Toll like receptors (TLR) 2 and 4 present on innate immune cells of the dental pulp detect cariogenic bacteria. Along with bacteria, *C. albicans* may also be present in dental caries. The presence of *C. albicans* can be detected by Dectin-1 a C type Lectin receptor. Expression of Dectin-1 in human pulpits has not been reported. Similarly, cytokines are released as a consequence of dental pulp inflammation caused by cariogenic bacteria. The T helper (Th) 1 inflammatory response leads to exacerbation of inflammation and its relationship with Osteopontin (OPN) is not known in pulp inflammation.

**Objective:**

The aim of this study was to observe the expression of Dectin-1, TLR-2, OPN and pro-inflammatory cytokines in irreversibly inflamed human dental pulp and to observe relationship between Dectin-1/TLR-2 and OPN/Pro-inflammatory cytokines in the presence of appropriate controls.

**Methods:**

A total of 28 subjects diagnosed with irreversible pulpitis were included in this ex-vivo study. Fifteen samples were subjected to standard hematoxylin and Eosin (H&E) and immunohistochemistry staining. Whereas, gene expression analysis was performed on 13 samples to observe mRNA expression of pro-inflammatory cytokines; tumor necrosis factor-alpha (TNF-α), interleukin (IL)-1 beta (ß), IL-6 Dectin-1, OPN, TLR-2 and TLR-4. SPSS version 21 was used for statistical analysis. One way analysis of variance (ANOVA), Pearson correlation and Chi-square test were used at *p* ≤ 0.05.

**Results:**

Gene expressions of Dectin-1, TLR-2 and TLR-4 were observed in all samples. Dectin-1 and TLR-2 expressions were significantly correlated (r = 0.5587, *p* = 0.0002). Similarly, OPN and TNF-α expression showed a significant correlation (r = 0.5860, *p* = 0001). The agreement between histologic and clinical diagnosis was 69.2% in the cases of irreversible pulpitis.

**Conclusion:**

Dectin-1 was expressed by inflamed human dental pulp. Dectin-1 and TLR-2 expression pattern was suggestive of a collaborative receptor response in inflamed pulp environment. OPN and TNF-α expressions showed a positive correlation indicating a possible relationship.

## Background

Pulpitis is inflammation of the dental pulp caused by cariogenic micro-organisms, trauma, or faulty restorations [1]. Primary cariogenic micro-organisms include *Streptococcus mutans (S. mutans)* and *lactobacilli* [2, 3]. *Candida albicans (C. albicans)*, a commensal of the oral cavity, may co-colonize carious lesions along with *S. mutans* [4–7]. Toll like receptor (TLR) -2 and 4 detect gram-positive and gram-negative bacteria, respectively. These receptors have been extensively reported previously in the dental literature for their role in inflammation of dental pulp and periapex [8–12]. *C. albicans* is detected by a pathogen recognition receptor (PRR) known as Dectin-1, a C-type lectin (CLR) [13, 14][15]. However, limited information is available in the dental literature about the role of Dectin-1 and other C-type lectins in dental pulp inflammation.

A couple of literature reviews have reflected on the role of CLRs in pulp inflammation, however, both these reviews were deficient in providing any scientific evidence for this claim. [16, 17]. While Yoo et al. discussed the role of CLRs in endodontic infections and specifically mentioned Dectin-1, his review also lacked citations from any investigative study [18]. Harmon examined and discovered the expression of DC-SIGN, another CLR situated on dendritic cells beneath human carious dentin [19]. To the best of our knowledge, the literature appears deficient in investigations of Dectin-1 expression in human pulpitis. This presents as a significant knowledge gap in the scientific literature and creates a need to address it.

Recent studies have described the clustering of TLRs and CLRs towards fungal pathogens. More specifically, collaborative signaling of TLR-2 with Dectin-1 is required for release of TNF-α, IL-1β and IL-6 [20–23]. In the absence of this collaboration, the T helper cell (Th)-1 inflammatory response can be deficient [24]. Since TLR-2 is an active participant in pulp inflammation, the role of this collaborative signaling also needs to be explored in inflamed dental pulp [8–12].

Pulp inflammation is a complex phenomenon initiating from the release of cytokines from inflammatory cells such as macrophages, monocytes, and neutrophils [25]. These cytokines up, or down regulating other inflammatory pathways and activation of inflammatory cells. TNF-α is an inflammatory mediator that is active in Th1 response. It is closely linked to the expression of Osteopontin (OPN) [24].

Other cytokines involved in the regulation of pulp inflammation include IL1-ß and IL-6 [26]. IL-6 performs both pro and anti-inflammatory actions. In its anti-inflammatory role, it causes Th2 polarization by inhibiting interferon gamma (Inf-γ) [27]. Its pro-inflammatory roles are mediated though the activation of the Th17 response [28]. IL-1ß causes upregulation of IL-8 and subsequent recruitment of neutrophils to the site of inflammation [29]. Activation of the Th1 response is mediated by Inf-γ and results in progression of inflammation [30]. On the other hand, the Th2 response is mediated by TGF-ß and results in downregulation of inflammation [31]. A prior study has shown that an effective pulp response towards invading pathogens will involve both Th1 and Th2 responses [32]. This essentializes the role of innate immune responses. in pulp inflammation and with OPN regulating the Th1 response, warrants further investigation with respect to human pulpitis [33, 34].

Considering the knowledge gaps identified in previous paragraphs, the aim of this ex-vivo study was to, (1) Observe and correlate expression of Dectin-1 and TLR-2 by gene expression in irreversibly inflamed human dental pulp, and (2) observe and correlate gene expressions of TNF-α, IL1-ß, IL-6 and OPN. The null hypothesis was that the gene expression of Dectin-1 and OPN are not elevated in inflamed human dental pulp when compared to appropriate controls.

## Methods

### Study design

**Fig. 1:**
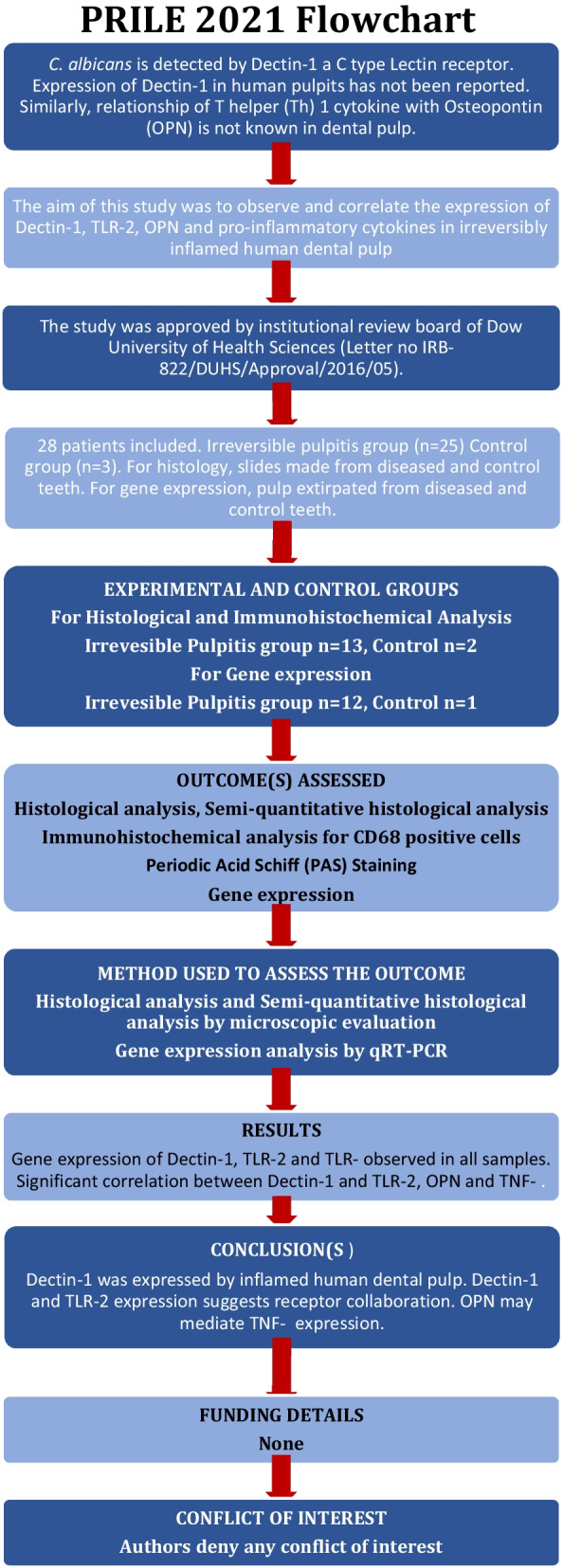
Study design according to PRILE guidelines

This manuscript was prepared in accordance with the Preferred Reporting Items for Laboratory studies in Endodontology (PRILE) 2021 guidelines (Fig. 1) [35]. This ex-vivo study was approved by the institutional review board of Dow University of Health Sciences (Letter no IRB-822/DUHS/Approval/2016/05). All human participation was as per Helsinki declaration; participation was voluntary, and selected participants provided informed consent. A total of 28 patients were included from the Oral and Maxillofacial Department and Department of Operative Dentistry, Dow Dental College, Dow University of Health Sciences. Systemically healthy patients presenting with clinical symptoms of irreversible pulpitis in either a premolars and molars and who chose extraction as treatment of choice were recruited for the study. The diagnosis of irreversible pulpitis was made if a patient presented with a history of spontaneous and/or lingering pain, either unprovoked or provoked, due to thermal stimuli that may radiate to adjacent anatomical structures. The diagnosis was confirmed through an an intraoral examination and observing dental caries in a suspected tooth, performing cold test and observing the radiograph of the suspected tooth for presence of deep carious lesion involving pulp [36]. Only cases that developed pulpitis secondary to dental caries were included. Whereas, teeth that did not contain viable pulp tissue, presented with periapical lesions radiographically (PAI > 2) [37] associated with pulp necrosis, developed pulpitis secondary to dental trauma, had previously initiated endodontic treatment, were fractured or showed root resorption were excluded. The samples with low yield and/or purity of total RNA as determined by nanodrop were also excluded. Sound teeth without any restoration, caries or periodontitis, extracted for orthodontic purpose were used as control. Out of included 28 teeth, 15 were used in histology and 13 pulp samples were subjected to Quantitative Realtime Polymerase Chain Reaction.

### Sample size calculation

The sample size was calculated using Open Epi online sample calculation software [38]. Mean and standard deviation of IL-6 (Disease sample 36 pg/dL ± 3.6, Control 0.01 pg/dL ± 0.02) was used from a previously published paper [39]. At a confidence level of 95% a power of 80% and effect size of 10, a sample size of 1 per group was calculated.

### Specimen preparation for histology (Cases *n* = 13, Control *n* = 2)

The extracted teeth (*n* = 15) obtained from the patients were immediately placed in a 15 ml falcon containing 10% buffered formalin (Sigma-aldrich, St. Louis, Missouri, United States) for at least 24 h. To facilitate decalcification process, the teeth were decoronated at 2 to 3 mm below the cemento-enamel junction. Coronal parts of the decoronated teeth were transferred to a new 15 ml falcon tube containing 10% formic acid. Teeth were checked every 5th day for appropriate decalcification with the help of a no. 22 blade (Feather Surgical Blade, Feather Safety Razor Co Ltd., Osaka, Japan). If the blade cut the tooth it was considered an end point of the decalcification step. Otherwise, formic acid was replaced with a fresh batch liquid and process repeated until desired results were obtained. Once the decalcification was complete, the teeth were sectioned with a no. 22 blade into two halves to allow widest area of pulp to be observed. For instance, in a mandibular molar the sectioning was performed mesiodistally, while for maxillary premolars a buccolingual plane was selected. Both halves of the tooth were used. After tissue processing, 4 um sections were cut and placed on charged slides and viewed under compound microscope (Motic BA310, Motic Inc. Co. Ltd, Hongkong). Images were captured with the help of a microscope mounted high definition camera (Moticam, Motic Inc. Co. Ltd, Hongkong) and a proprietary software (Motic Images Puls 3.0, Motic Inc. Co. Ltd, Hongkong). Captured images were viewed on a standard LCD computer screen and observed for histological and immunohistochemical analysis.

### Histological analysis

For each of the selected samples, standard H&E staining was performed.

An assessment criteria previously described by Anderson [40] was used to histologically classify H&E slides. In summary, normal pulp was diagnosed if no reduction in size of the of the odontoblast layer or odontoblasts was noted and if the dentinal tubules were arranged parallel to each other without any organic (e.g. bacteria) or inorganic deposits occluding it. In addition, no dilated vessels, accumulation of inflammatory cells, bacteria or tissue edema were identified.

The diagnosis of reversible pulpitis (RP) was made according to following criteria; atrophic pulp (fewer fibroblasts and a reduced or flattened odontoblast layer), abundant tertiary dentin reducing the volume of pulp space, evidence of moderate inflammation predominant in lymphocytes and plasma cells confined to the coronal pulp (in some cases without tissue destruction) and absence of necrosis or bacteria.

The diagnosis of irreversible pulpitis (IP) was made according to the following criteria: (1) Presence of either partial or total pulp necrosis, (2) Evidence of liquefaction or coagulation of tissue surrounded by polymorphonuclear neutrophils (PMNs), (3) Peripheral concentration of chronic inflammatory cells (lymphocytes, plasma cells, and macrophages) around this central zone of liquefaction or coagulation and (4) Presence of bacteria is observed either within the pulp or in the dentinal tubules. A direct communication between dental caries front and pulp chamber may be present.

The histologic diagnosis of acute/chronic inflammation was made according to the criteria described by Giuroiu [41]. For acute inflammation perivascular infiltration of PMNs with associated degeneration of odontoblasts, vascular congestion and edema were noted. In chronic pulpitis, chronic inflammatory infiltrate, diffuse calcifications, extravasated red blood cells, fibrous tissue formation and preservation of odontoblasts was identified.

### Semi quantitative histological analysis

The inflammatory parameters were quantified according to the criteria set forth by Bruno et al. [42]. This is summarized below.

#### Intensity

Intensity of inflammatory infiltrate was scored as either absent, mild or intense. It was scored for each specimen at high magnification (× 100).

Scoring for intensity was performed as follows:

- No inflammation (0), when specimen area had no inflammatory cells;
- Mild inflammation (1), when < 35% of specimen area was filled by inflammatory cells;
- Intense inflammation (2), when more than 35% of specimen area was filled by inflammatory cells.

#### Collagen deposition

It was characterized by an eosinophilic area with reduced cellularity and blood vessels density or an even acellular eosinophilic region lacking blood vessels.

The collagen deposition was scored as follows:

- Mild (1), when < 35% of specimen area was hyalinized
- Intense (2), when more than 35% of specimen area was hyalinized.
- Absent (0), when specimen area showed almost no collagen due to extensive tissue destruction

*Calcification* The calcification was scored as absent (0), mild (1), and intense (2).

*Necrosis* The necrotic areas were considered as absent (0) or present (1).

A final score was calculated as the sum of these four score values.

### Immunohistochemical analysis

Immunohistochemical analysis for CD68 was performed on all 15 histology specimens. Paraffin embedded unstained slides were incubated in thermal block (HB-100, Bioer Technology Co, Ltd, China) for 30 min at 70 °C. Antigen unmasking was performed with Tris–HCL solution (Invitrogen, USA) at 100 °C for 30 min followed by washing with wash buffer (Phosphate buffered saline + Tween) (Sigma-aldrich, St. Louis, Missouri, United States). This was followed by blocking of antigen with 3% Hydrogen Peroxide for 5 to 10 min to prevent non-specific binding. Blocking solution was later washed off with a washing buffer. Primary antibody (CD68^+^ Pre-diluted, Dako # 609, Agilent, Santa Clara, USA) was added and incubated at 37 °C for 1 h. Primary antibody was washed off and horseradish peroxidase labelled secondary antibody (Abcam, Goat pAb to Ms IgG) was added similarly. Wash buffer was again used to remove any remaining secondary antibody. This was followed by application of 3,3′-Diaminobenzidine chromogen for 5 to 10 min, hematoxylin was added as a counter stain. Slides were than washed with distilled water and allowed to air dry. Lastly, the slides were preserved with Dibutylphthalate Polystyrene Xylene, mounting media and observed under compound microscope (Motic BA310, Motic Inc. Co. Ltd, Hongkong). Images were captured with the help of a microscope mounted on high definition camera (Moticam, Motic Inc. Co. Ltd, Hongkong) and a proprietary software (Motic Images Puls 3.0, Motic Inc. Co. Ltd, Hongkong).

### Periodic acid schiff (PAS) staining

PAS staining was performed for all selected teeth to identify *Candida* spp. The sections were deparaffinized and hydrated with distilled water. The sections were oxidized using 0.5% periodic acid solution for 5 min. The slides were rinsed with distilled water and immersed in Schiff agent for 15 min. This is when the sections on the slide appear pink. Following rinsing with warm water the sections are counter-stained with hematoxylin for 1 min, rinsed, dried and covered. The candida species appeared purple in a background of blue stained tissue during the microscopic examination.

### Quantitative real-time polymerase chain reaction: (Cases *n* = 12, Control *n* = 1)

**Fig. 2:**
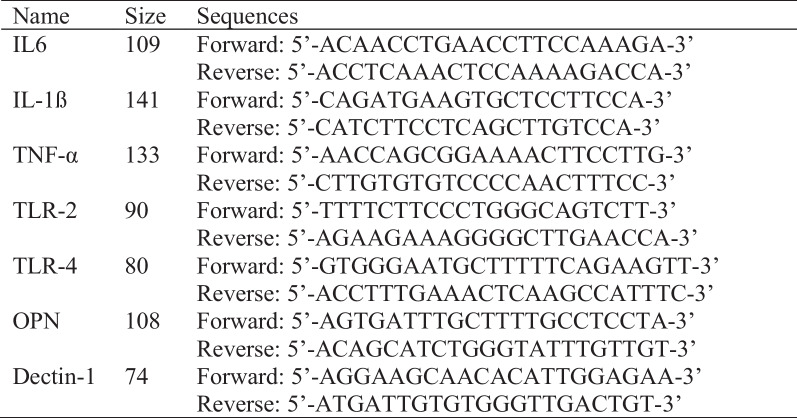
Primer sequence

All pulp sample were collected under rubber dam isolation. The tooth and rubber dam were disinfected with 2% Chlorhexidine Gluconate. Standard access cavity was prepared as per tooth type and location of dental decay, while avoiding damage to pulp tissue. After ensuring complete deroofing, working length was measured 1 mm short of the apex with the electronic apex locator (Root ZxII, J Morita, Tokyo, Japan). Using two K type files (Mani, Japan) in a braiding motion, the pulp was removed from the root canal and placed immediately into an Eppendorf tube containing Phosphate Buffered Saline. This sample was stored in a -80 freezer until further use. Later, the Eppendorf containing the pulp sample was allowed to thaw at room temperature. The pulp sample was shifted to an Eppendorf containing Trizol and homogenized using a homogenizer (Omni Mixer Homogenizer, Omni International, Georgia USA). Total RNA from pulp tissue was obtained by using the standard Trizol protocol [43]. Reverse transcription was performed according to the instruction manual of ThermoScientific RevertAid First strand cDNA Synthesis Kit (Thermo Fisher Scientific, Baltics UAB, Vilnius, Lithuania, Catalog no K1622). Primers used in the study were designed by using Primer-Blast NCBI, an online primer designing tool [44]. (Fig. 2) The primers were synthesized by Macrogen, Seoul, South Korea. Quantitative Real time Polymerase Chain reaction (qRT-PCR) was performed on QuantStudio 7 Flex (Thermo Fisher Scientific, Waltham, Massachusetts, United States) using 5 μl mixture of Maxima SYBR Green/ROX qPCR Master Mix (Thermo Fisher Scientific, Waltham, Massachusetts, United States) and cDNA of samples and 5 μl of primer in each well. Reaction mixtures were denatured for 10 min at 95 °C. Forty cycles of PCR were performed as follows: cyclic denaturation for 10 s at 95 °C, annealing for 1 min at 60 °C and extension for 1 min at 72 °C. ß-actin a housekeeping gene was used as an internal control for gene expression. The Δ ΔCT method was used to detect the fold change in gene expression.

### Statistical analysis

SPSS version 21 for Mac was used for statistical analysis. Descriptive statistics were used to analyze the basic study data. The semi-quantitative histological analysis was statistically analyzed using Chi-Square test. One way analysis of variance was used to compare means of fold change among the pulpitis samples. Pearson correlation was used to observe linear relationship between target genes. All analysis was performed at *p* ≤ 0.05. Shapiro–wilk test was performed to observe the normal distribution of data. Since the data was found to be normally distributed, parametric tests were performed.

## Results

A total of 34 samples were included in the study; 17 each for histology and qRT-PCR. Two samples were discarded in the histology group (necrotic pulp *n* = 1, failure to decalcify *n* = 1). In addition, 4 samples were discarded in the gene expression group (necrotic pulp *n* = 2, low yield/purity of RNA *n* = 2). Subsequently, only 28 samples were used for the study; 15 samples (2 control 13 study) were histologically analyzed, whereas 13 (1 control and 12 study) were included in the gene expression analysis.

### Histological analysis

**Fig. 3:**
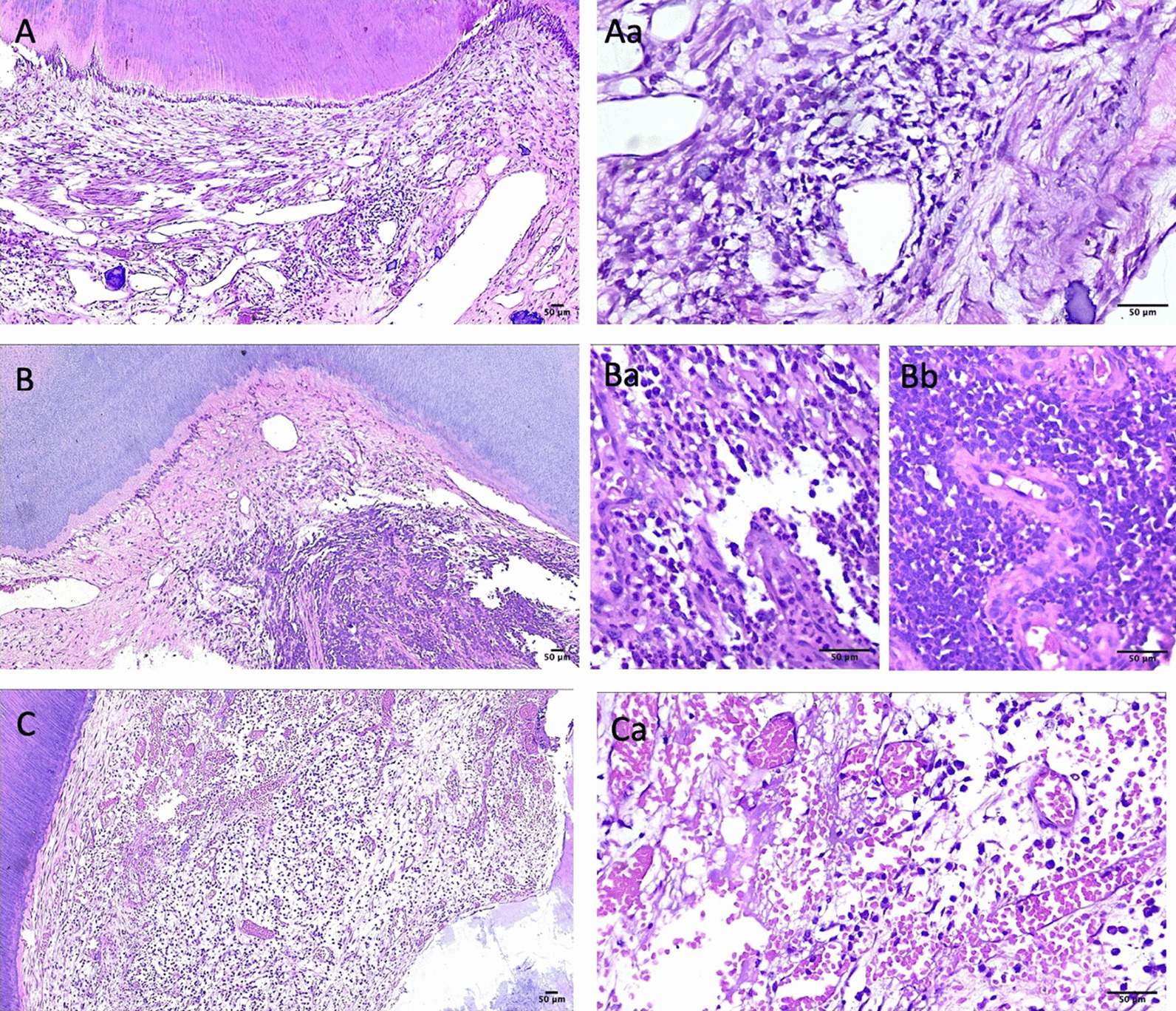
Histologic images of Irreversible Pulpitis of 3 representative samples. **A** Histologic image of sample ‘A’ diagnosed as reversible pulpitis (10x). **Aa** Image shows vascular dilation and inflammatory cells aggregation (40x). **B** Histologic image of sample ‘B’ diagnosed as Irreversible pulpitis (10x). **Ba**, **Bb** Image shows plasma cells, neutrophils and lymphocytes (40x). **C** Histologic image of sample ‘C’ diagnosed as Irreversible pulpitis (10x). **Ca** image show hyperemic pulp and red cell hemorrhage (40x). (scale bars = 50 um). Image adjusted for brightness and contrast

The diagnosis of irreversible pulpitis was made in 9/13 samples (69.2%) (Table 1). Thus, the histologic diagnosis corresponded with the clinical diagnosis in only 69.2% of the cases. Inflammatory infiltrates were found in all samples (Fig. 3A). Majority of the cell infiltrate was chronic in nature (7/13). Granulation tissue contained large quantities of blood vessels, fibroblasts and inflammatory cells. Lymphocytes, plasma cells and neutrophils were predominantly identified. Plasma cells were identified on the basis of their eccentric nuclei and purple cytoplasms, while neutrophils exhibited nuclei with multiple lobes (Fig. 3B,C). Pulp necrosis was noted in in 9/13 samples (69.2%, *p* = 0.000)). Edema and fibrosis were observed with higher frequencies.

**Table 1:** Descriptive Statistics

Microscopic feature		Number of cases	%
Intensity of	No	0	0
Inflammatory	Mild	8	61.5
infiltrate	Intense	5	38.5
Collagen Deposition	Absent	6	46.2
	Mild	6	46.2
	Intense	1	7.7
Calcification	Absent	6	46.2
	Mild	6	46.2
	Intense	1	7.7
Pulp Necrosis	Present	9	69.2
	Absent	4	30.8
Pulpitis	Reversible	4	30.8
	Irreversible	9	69.2
Type of	Acute	6	46.2
inflammation	Chronic	7	53.8
Macrophages	Scattered	2	15.4
	Positive	9	69.2
	Intense	2	15.4
Final Score	1	2	15.4
	2	3	23.1
	3	4	30.8
	4	2	15.4
	5	2	15.4

**Fig. 4:**
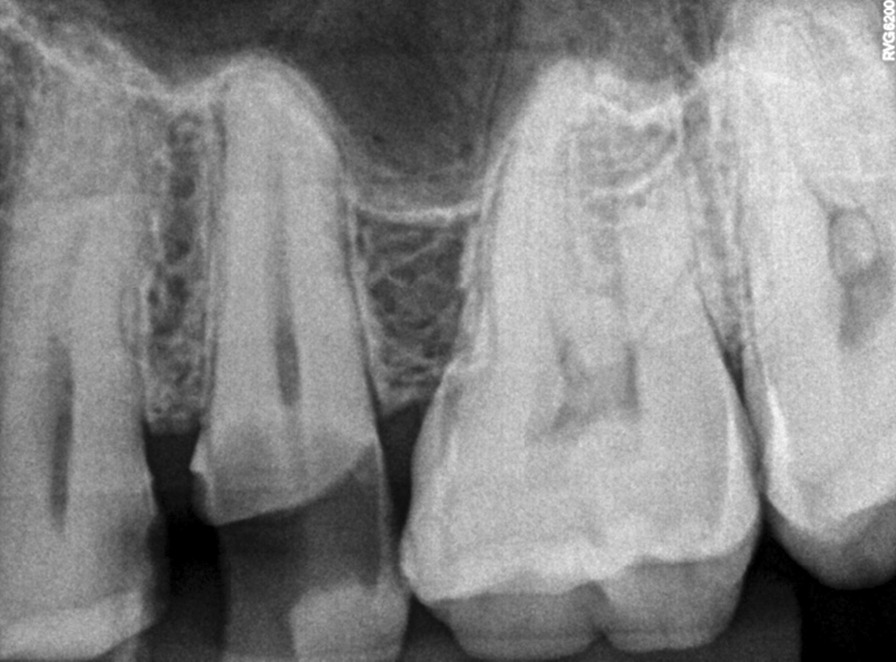
Radiograph of the tooth represented in Fig. 2A as sample ‘A’

All samples exhibited staining of a few cells with CD68. This positivity is expected to highlight macrophages. There were 2 samples that showed a higher population of cells staining positive for CD68 in comparison to the rest. These were situated beneath the odontoblast layer, near the site of carious exposure. Figure 4 shows radiograph of the tooth represented in Fig. 3A as sample ‘A’.

### Semi-quantitative histological analysis

The semi-quantitative analysis is presented in Table 2. An intense inflammatory infiltrate was noted in 4 samples with histological diagnosis of irreversible pulpitis versus only 1 in reversible pulpitis. Pulp necrosis was observed in all samples diagnosed as irreversible pulpits (9/9) and in none of the samples of reversible pulpitis (4/4) (*p* = 0.000). A milder infiltrate of chronic cells was statistically significant in samples with chronic inflammation (6/7) (*p* = 0.086). Features of acute inflammation were more common in samples with a histological diagnosis of reversible pulpitis, whereas samples representing histological diagnosis of irreversible pulpitis presented with features of chronic inflammation more frequently.

**Table 2:** Semi-Quantitative Analysis of type of Pulpitis and Inflammation with various Inflammatory Parameters

		Type of Pulpitis *n*(%)			*P value*	Type of Inflammation n(%)			*P value*
		Reversible	Irreversible	Total		Acute	Chronic	Total	
Inflammatory	Mild	3(23.1)	5(38.5)	8(61.5)	0.498	2(15.4)	6(46.2)	8(61.5)	0.086
Infiltrate	Intense	1(7.7)	4(30.8)	5(38.5)		4(30.8)	1(7.7)	5(38.5)	
Collagen	No	2(15.4)	4(30.8)	6(46.2)	0.786	4(30.8)	2(15.4)	6(46.2)	0.321
Deposition	Mild	2(15.4)	4(30.8)	6(46.2)		2(15.4)	4(30.8)	6(46.2)	
	Intense	0(0)	1(7.7)	1(7.7)		0(0)	1(7.7)	1(7.7)	
Pulp	Absent	1(7.7)	5(38.5)	6(46.2)	0.243	3(23.1)	3(23.1)	6(46.2)	0.450
Calcification	Mild	2(15.4)	4(30.8)	6(46.2)		2(15.4)	4(30.8)	6(46.2)	
	Intense	1(7.7)	0(0)	1(7.7)		1(7.7)	0(0)	1(7.7)	
Pulp Necrosis	Absent	4(30.8)	0(0)	4(30.8)	0.000*	2(15.4)	2(15.4)	4(30.8)	0.853
	Present	0(0)	9(69.2)	9(69.2)		4(30.8)	5(38.5)	9(69.2)	
Macrophages	+	1(7.7)	1(7.7)	2(15.4)	0.532	0(0)	2(15.4)	2(15.4)	0.359
	+ +	3(23.1)	6(46.2)	9(69.2)		5(38.5)	4(30.8)	9(69.2)	
	+ + +	0(0)	2(15.4)	2(15.4)		1(7.7)	1(7.7)	2(15.4)	
Final Score	1	0(0)	2(7.7)	2(7.7)	0.406	1(7.7)	1(7.7)	2(15.4)	0.369
	2	2(15.4)	1(7.7))	3(23.1)		1(7.7)	2(15.4)	3(23.1)	
	3	1(7.7)	3(23.1)	4(30.8)		2(15.4)	2(15.4)	4(30.8)	
	4	1(7.7)	1(7.7)	2(15.4)		2(15.4)	0(0)	2(15.4)	
	5	0(0)	2(15.4)	2(15.4)		0(0)	2(15.4)	2(15.4)	

*p* value calculated using chi square test. *p* ≤ 0.05 is significant

*indicates significant value

### Immunohistochemical analysis

**Fig. 5:**
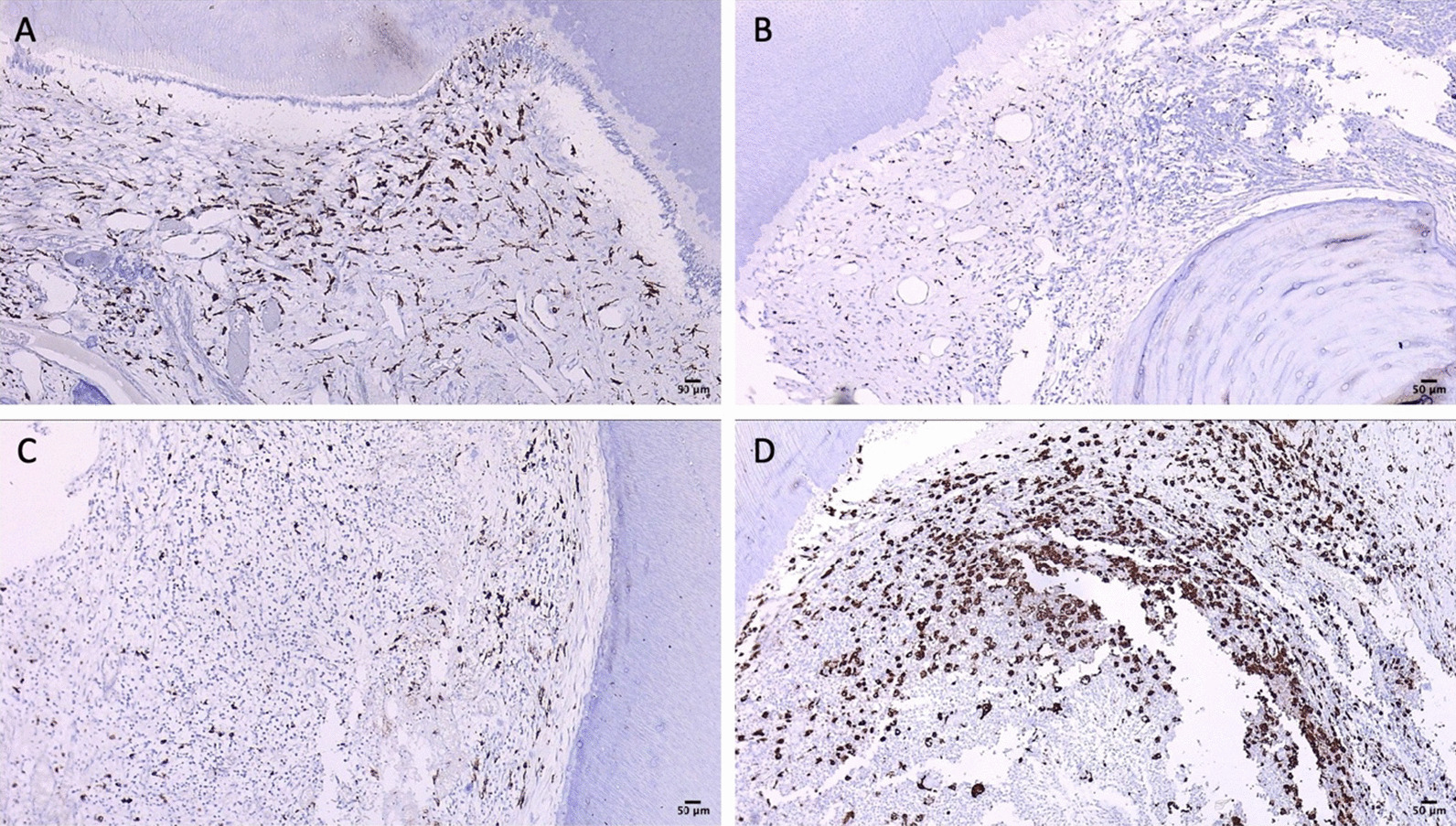
CD68 expression by Immunohistochemistry. **A**, **C** and **D** Accumulation of macrophages beneath odontoblasts (40x). **B** Sparse expression of macrophages in a case of chronic inflammation (40x). (scale bars = 50 um). Image adjusted for brightness and contrast

As mentioned in the previous section, the CD68 + ve macrophages were observed in all samples. In control sample there were few macrophages scattered throughout the pulp tissue. However, in the disease samples, the number of macrophages drastically increased in number. The intensity of macrophage infiltration was particularly strong in the pulp below the odontoblast layer. (Fig. 5).

### PAS analysis

**Fig. 6:**
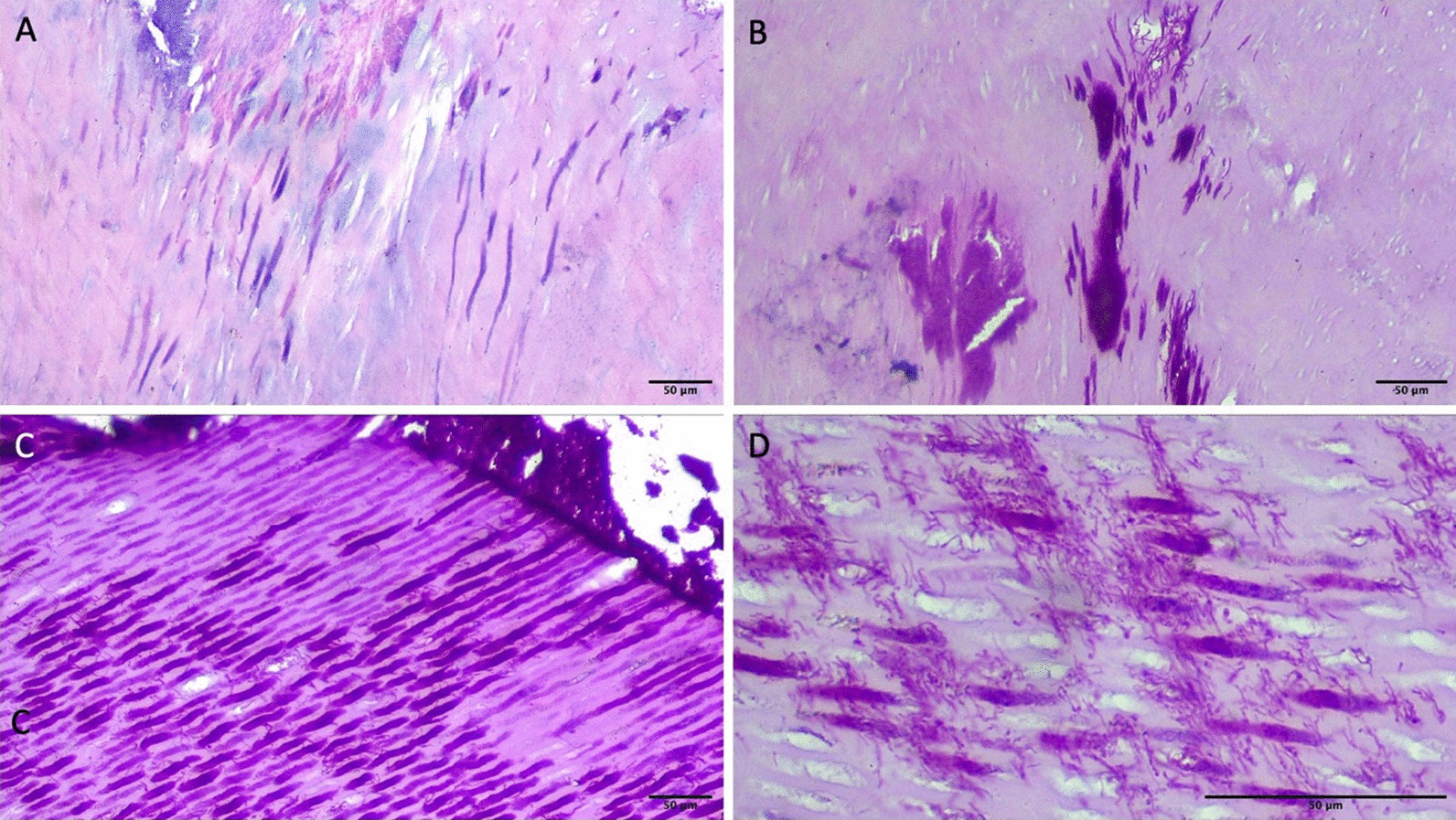
PAS staining. **A** H&E staining of carious dentin showing microorganisms in the dental tubules (40x). **B** PAS staining of same sample as in Fig. 5A shows that the PAS stain follows the pattern of H&E stain (40x). **C** PAS stain showing potential microorganisms in dentinal tubules (40x). **D** High magnification (100x) image of Fig. 5C. (scale bars = 50 um). Image adjusted for brightness and contrast

The PAS staining failed to identify any *C. albicans* within the pulpal tissue. The PAS staining generally followed the pattern of H&E staining (Fig. 6).

### Gene expression analysis

**Fig. 7:**
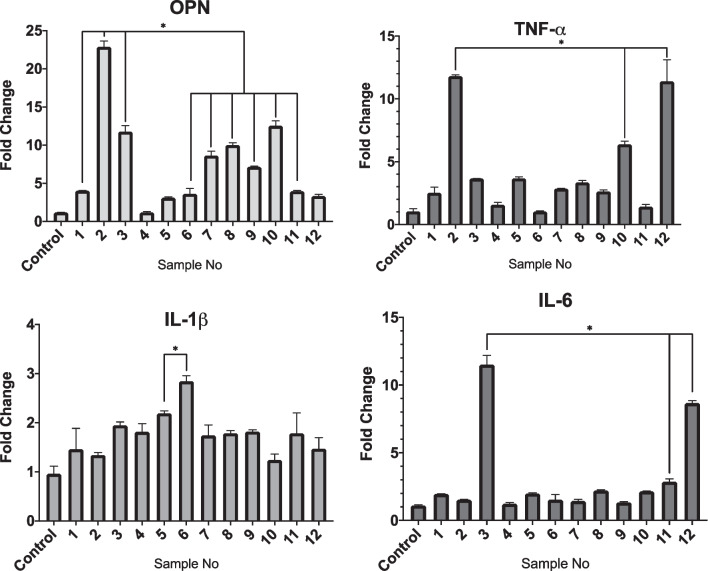
Relative expression of IL-1ß, IL-6, TNF-α and OPN, in clinical samples. The significant differences (*p* ≤ 0.05) are indicated by *. *p*-value calculated using ANOVA and post hoc Tukey’s test

**Fig. 8:**
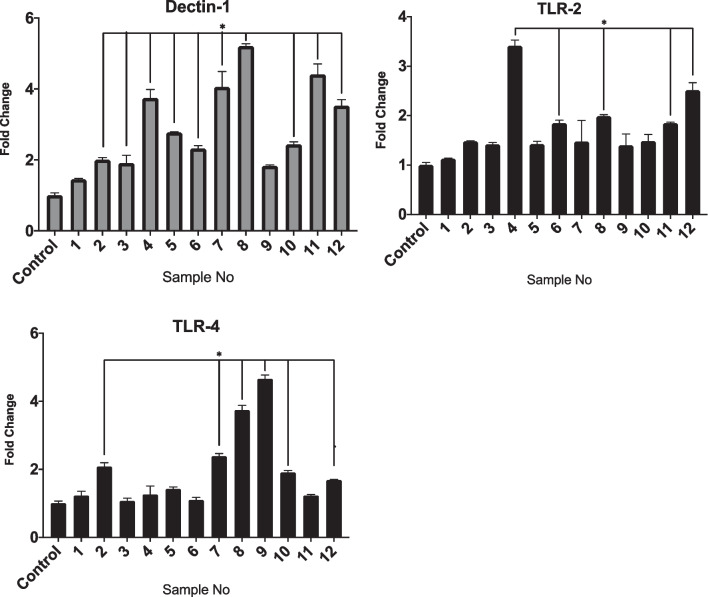
Relative expression of Dectin-1, TRL-2 and TLR-4 in clinical samples. The significant differences (*p* ≤ 0.05) are indicated by *. *p*-value calculated using ANOVA and post hoc Tukey’s test

The expressions of all inflammatory markers IL-6, IL-1β and TNF-α were elevated in comparison to the control (Fig. 7). However, significant differences were noted in only a few samples as compared with controls. For instance, significant expression of TNF-α was found in sample 2 (11.8 folds), 10 (6.3 folds) and 12 (11.3 folds) (Fig. 5). The OPN was similarly expressed in all samples with significant expression in samples 1 (4 folds), 2 (22.8 folds), 3 (11.7 folds), 6 (3.6 folds), 7 (8.6 folds), 8 (10 folds), 9 (7.1 folds), 10 (12.5 folds) and 11 (3.9 folds). Dectin-1, TLR-2 and TLR-4 (Fig. 8) were found to be raised in all samples. Sample numbers 4, 6, 8, 11 and 12 showed statistically significant expressions for both Dectin-1 and TLR-2 as compared to control. Generally, Dectin-1 was observed to be expressed at an elevated level when compared to TLR-2 and 4.

### Correlational analysis

**Fig. 9:**
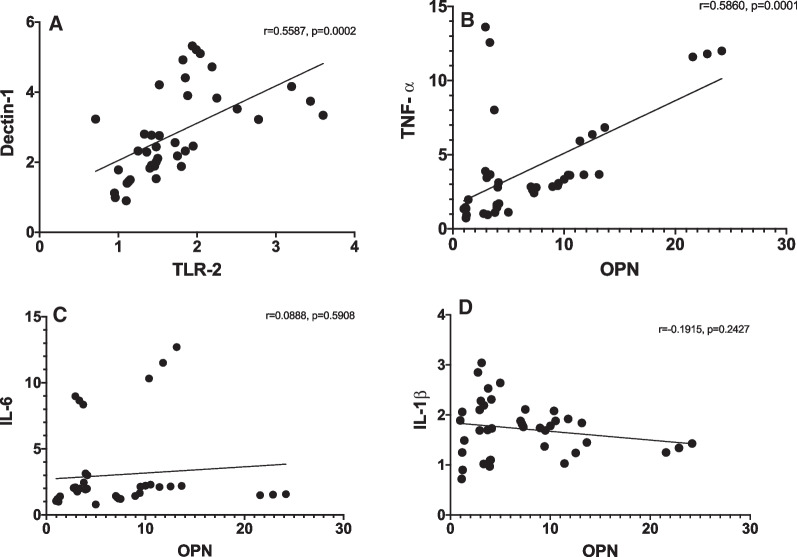
Correlational analysis. Pearson correlations at *p* ≤ 0.05. Each graph contains Pearson’s r and respective *p* value on top right corner. **A** Dectin-1 and TLR-2, **B** OPN and TNF-a, **C** OPN and iL-6, **D** OPN and IL-1b

A significant positive correlation (r = 5587, *p* = 0.0002) was observed between TLR-2 and Dectin-1(Fig. 9). A similar significant positive correlation (r = 0.586, *p* = 0.0001) was found between OPN and TNF-α. However no correlation was observed between OPN-IL6 (r = 0.0888, *p* = 5908) and OPN-IL-1β (r = -0.1915, *p* = 0.2427).

## Discussion

Our study is perhaps the first to report the expression of Dectin-1 in human dental pulp. The results of our study indicate that the immunocompetent cells of inflamed human dental pulp express Dectin-1. We also found that the expression of TLR-2 was positively correlated with that of Dectin-1 and may also demonstrate the polymicrobial nature of dental caries. The expression levels of OPN and TNF-α, a Th1 cytokine also showed positive correlation, implicating a potential relationship between the two. We failed to identify any fungal pathogens through PAS staining in carious lesions or within the dentinal tubules. Our results corroborate the findings of our animal study[45] in which we observed similar findings and in addition also observed Dectin-1 expression in the macrophages located within the inflamed pulp of mice through immunohistochemistry.

Dental caries is a dynamic process mainly caused by gram-positive bacteria; *S. mutans* and *Lactobacillus* spp. TLR-2 is responsible for detection of gram-positive bacteria by detecting the lipoteichoic acid, peptidoglycans, and lipopeptides [46]. Similarly, TLR-4 detects gram-negative micro-organisms by recognizing their lipopolysaccharides (LPS) [47]. Historically, it has been shown that these two receptors play a central role in dental pulp innate immunity [11, 12, 46–49]. While our results partially corroborate the previously published research, our findings indicate that Dectin-1 is also actively expressed along-with the TLRs, and may complement them in providing an adequate innate immune response.

Dectin-1 is expressed on macrophages and dendritic cells. These cells along with odontoblasts are the first line of defense against any invading pathogen. The activation of the type of receptor depends upon the pathogen associated molecular pattern (PAMP). While dental caries is initiated by gram positive microorganisms, progressive and deeper layers of dental caries harbor more gram-negative microorganisms, such as *Prevotella intermedia* and *Porphyromonas endodontalis* [50–52]. Also, as described earlier, co-colonization with *C. albicans* may also occur. This polymicrobial biota initiates a myriad of signaling pathways within the dental pulp. Therefore, it is logical to assume that every carious lesion may have a different ecology with unique of PRRs. Our results concur with this explanation. In sample 8, TLR-2 (2 folds) and 4 (4 folds) both were raised indicating the presence of gram-positive and gram-negative organisms. Samples 4, 6, 8, 11 and 12 presented with elevated levels of Dectin-1 (3.7, 2.3, 5.2, 4.4 and 3.5 folds respectively) and TLR-2 (3.4, 1.8, 2, 1.8 and 2.5 folds respectively), indicating a combined fungal and gram-positive ecology. Sample 9 presented with raised expression of TLR-4 (4.6 folds) only, suggesting an exclusive gram-negative ecology, characteristic of deep carious lesions. These results provide a preliminary evidence that inflamed dental pulp expresses Dectin-1 in response to exposure to polymicrobial biota.

The positive linear relationship between TLR-2 and Dectin-1 (r = 5587, *p* = 0.0002) discovered in this investigation may be explained as collaborative in nature. A prior study has shown that signaling pathways generated by both these receptors are necessary for an adequate innate immune response against a fungal pathogens [23]. This collaborative signaling results in release of TNF-α, IL-1β and IL-6 [20–23]. Although CLRs are specific for fungal pathogens, TLRs can also detect fungal organisms. TLR-2 recognizes phospholipomannan, while TLR-4 may detect O linked-mannan present in the cell wall of *C. albicans* [53–55].

Our results showed a significant correlations between the expression of OPN and TNF-α, a th1 cytokine. The expression of TNF-α in inflamed pulp environment has been described before. Galicia [56] observed upregulation of TNF-α in pulpitis. Pezelj-Ribaric reported that expression of TNF-α differs between symptomatic and asymptomatic irreversible pulpitis and can be a potential biomarker for determining severity of pulpitis [57]. Our results verify these findings. However, these studies have observed the expression of TNF-α in pulpitis without exploring the regulation pathways. Our results suggest that OPN may mediate TNF-α expression. The relationship of OPN and TNF-α has been described previously [58, 59].

OPN is a pleotropic molecule with diverse functions [60]. OPN is produced by macrophages, dendritic cells, polymorphonuclear leukocytes and T- and B- lymphocytes [61, 62]. OPN released by pulp dendritic cells plays an important role in odontoblast differentiation [63]. OPN production results in upregulation of Interleukin-12, TNF-α and subsequent Th1 and Th17 responses [33, 64]. This leads to recruitment of macrophages, dendritic cells, polymorphonuclear leukocytes and T- and B- lymphocytes. The T-cells produce Interferon Gamma (INF-γ), a potent modulator of Th1 polarization [24]. OPN knockout animals were found to have a deficient Th1 response [33, 34]. Therefore, OPN is essential for an optimal Th1 response.

The relationship of OPN and other inflammatory cytokines, IL-6 and IL-1β was not statistically significant. IL-6 is a pleotropic molecule produced by a variety of innate immunity cells. Farges reported that odontoblasts produce IL-6 on activation of TLR-2 [65]. It has both anti- and pro-inflammatory properties [66]. A study found raised level of IL-6 in inflamed dental pulp [39]. Another study found its expression to be independent of IL-1β expression when human dental pulp was challenged with LPS [67]. IL-1β is produced by many cell types and along-with IL-8, it mediates inflammatory response by recruiting PMNs [68]. IL-1β was found upregulated in pulpitis [56], 69. Another study showed that IL-1 producing cells were macrophages [70]. We also found raised expression of both of these cytokines; IL-6 and IL-1β. However, in contrast to TNF-α, the correlation of these cytokines was not significant with respect to OPN. It may be because histologically our samples were more diverse, consisting of both chronic and acute inflammatory infiltrate, and these cytokines play different roles in acute and chronic stages of inflammation. Since inflammation involves overlapping networks of pro and anti-inflammatory events [71], it is logical to assume that the levels of these cytokines with diverse functions may not coincide with OPN. Additionally, OPN may regulate TNF-α but not IL-6 and IL-1β in an inflamed dental pulp environment, which may explain lack of significant correlation.

PAS staining failed to show any *C. albicans*. These results agree with a prior investigation by Maijala [72]. While the pattern of PAS staining was consistent with the H&E staining, no *C. albicans* were noted within the dentinal tubules. The purpose of our study was not to assign an etiological role to *C. albicans*, rather to see if it is recognized by the innate immunity of dental pulp. *C. albicans* has been identified in carious lesions by some studies [73–76]. However, its etiologic potential in dental caries has yet to be proven [72]. Currently, only preliminary evidence exists [77, 78]. Recent studies have suggested a possible co-colonization of *C. albicans* with cariogenic bacteria. It has the ability to bind to *S. mutans*–derived glucosyltransferase B (GtfB) thus enhancing its virulence (18, 19). Recent evidence also points towards a strong association between severe early childhood caries and coinfection with *C. albicans* and *S. mutans* [79–82]. We believe that additional work is required to understand the role this co-colonization might play in increasing the virulence and caries severity.

The histological diagnosis of our samples agreed with the clinical diagnosis in 9/13 samples (69.2%). These results are consistent with Giuroiu’s finding who found a 68.62% agreement between clinical and histological diagnoses [41]. However, our results are inferior to the 84% agreement between the clinical and histologic findings reported by Ricucci [83]. Similarly, another study found a kappa agreement of 0.843 between clinical and histologic diagnosis [84]. These differences can be attributed to variations in methodology, study design, patient characteristics, diagnostic criteria and various other factors that are difficult to standardize.

In conclusion, our study provides preliminary data on gene expression of Dectin-1, a β-glucan receptor in inflamed human dental pulp. More studies are required to identify the different cell types of dental pulp expressing this receptor and it will be interesting to know if odontoblasts express it. Additionally, the collaborative relationship of Dectin-1 with TLR-2 in dental pulp inflammation was identified. However, this warrants further investigation owing to a polymicrobial nature of dental caries. The potential role of OPN in regulating Th-1 inflammatory response in dental pulp inflammation also requires further investigation and can be an area of research interest with potential therapeutic importance. According to the results of our study, the null hypothesis was rejected. The limitations include a limited sample size, and lack of confirmation of Dectin-1 expression by immunohistochemical analysis and/or protein expression analysis.

## Conclusions

Dectin-1 was expressed by inflamed human dental pulp. Dectin-1 and TLR-2 expression showed a positive correlation that is suggestive of a collaborative receptor response in and inflamed pulp environment. The expression pattern of OPN and TNF-α showed a positive correlation suggesting a possible relationship between the two cytokines.

## Data Availability

All data that was generated or analyzed during this study are included in this article.
